# Preparation of Novel *meta-* and *para-*Substituted *N*-Benzyl Protected Quinuclidine Esters and Their Resolution with Butyrylcholinesterase

**DOI:** 10.3390/molecules17010786

**Published:** 2012-01-16

**Authors:** Ines Primožič, Marijana Bolant, Alma Ramić, Srđanka Tomić

**Affiliations:** Department of Chemistry, Faculty of Science, University of Zagreb, Horvatovac 102A, HR-10 000 Zagreb, Croatia

**Keywords:** quinuclidin-3-ol, esters, resolution, butyrylcholinesterase, *N*-benzyl protected groups

## Abstract

Since the optically active quinuclidin-3-ol is an important intermediate in the preparation of physiologically or pharmacologically active compounds, a new biocatalytic method for the production of chiral quinuclidin-3-ols was examined. Butyrylcholinesterase (BChE; EC 3.1.1.8) was chosen as a biocatalyst in a preparative kinetic resolution of enantiomers. A series of racemic, (*R*)- and (*S*)-esters of quinuclidin-3-ol and acetic, benzoic, phthalic and isonicotinic acids were synthesized, as well as their racemic quaternary *N*-benzyl, *meta-* and *para*-*N*-bromo and *N*-methylbenzyl derivatives. After the resolution, all *N*-benzyl protected groups were successfully removed by catalytic transfer hydrogenation with ammonium formate (10% Pd-C). Hydrolyses studies with BChE confirmed that (*R*)-enantiomers of the prepared esters are much better substrates for the enzyme than (*S*)-enantiomers. Introduction of bromine atom or methyl group in the *meta* or *para* position of the benzyl moiety resulted in a considerable improvement of the stereoselectivity compared to the non-substituted compounds. Optically pure quinuclidin-3-ols were prepared in high yields and enantiopurity by the usage of various *N*-benzyl protected groups and BChE as a biocatalyst.

## 1. Introduction

**Figure 1:**
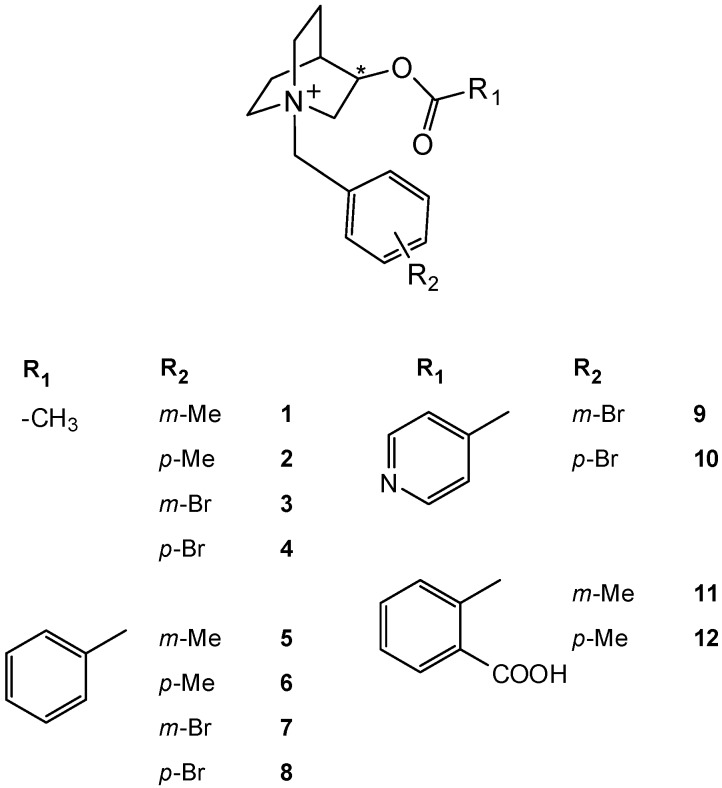
Synthesized quinuclidinium esters.

**Scheme 1:**
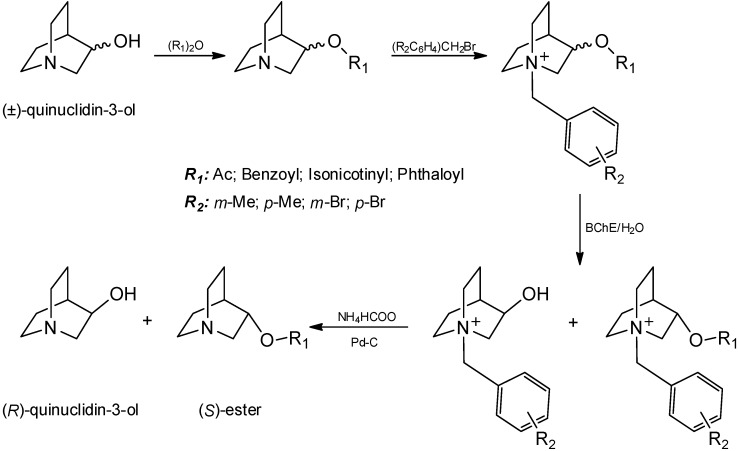
Syntheses of esters and the method for resolution with butyrylcholinesterase.

Butyrylcholinesterase (BChE) is a non-specific ester hydrolyzing enzyme with no known endogenous physiological substrate [1]. It can hydrolyze various esters of choline and other compounds [2] (e.g., cocaine, some organophosphorus compounds), and it is important because of several pharmacological and toxicological functions (e.g., as bioscavenger for the protection of humans against organophosphate toxicity [3], biomarker of exposure to toxic organophosphorus compounds in food and environment [4,5]). This enzyme has not been used to a large extent as a biocatalyst in organic chemistry mainly as a result of its affinity towards positively charged substrates [6]. Structural complementarity of choline and quinuclidin-3-ol pointed to racemic quinuclidine derivatives as candidates for kinetic resolution catalyzed by this enzyme [7,8,9]. Compounds which contain quinuclidin-3-ol moiety are valuable intermediates for the synthesis of optically active pharmaceuticals [10], thus, efficient synthesis of chiral quinuclidin-3-ols is necessary for the preparation of biologically active compounds. In our previous work, various chiral quaternary esters of quinuclidin-3-ol were subjected to hydrolysis in the presence of BChE [11], and it was shown that hydrolysis is highly stereoselective. Kinetic studies revealed the preference of BChE toward (*R*)-enantiomers of quinuclidin-3-ol compounds. Therefore, we decided to synthesized a series of new quaternary esters with *meta-* and *para*-bromo and *meta-* and *para*-methylbenzyl protecting groups at the quinuclidine nitrogen atom, to determine whether the substituent at the benzyl moiety can further enhance enantioselectivity of the hydrolysis. A series of racemic, (*R*)- and (*S*)- esters of quinuclidin-3-ol and acetic, benzoic, phthalic and isonicotinic acids were synthesized as well as their racemic quaternary *meta* and *para* *N*-bromo and *N*-methylbenzyl derivatives (Figure 1). After the kinetic resolution using BChE as a catalyst, all *N*-benzyl protected groups were successfully removed by catalytic transfer hydrogenation with ammonium formate (10% Pd-C, Scheme 1). The stereoselectivity of hydrolysis with horse serum BChE was investigated. A method for the preparation of chiral (*R*)-esters and (*S*)-quinuclidin-3-ol by the enzymic kinetic resolution on a preparative scale was proposed.

## 2. Results and Discussion

Twelve quaternary racemic esters of quinuclidin-3-ol and acetic, benzoic, isonicotinic and phthalic acids were synthesized, and a method for their resolution with butyrylcholinesterase was explored (Scheme 1). All esters were synthesized in good yields by the esterification reaction of quinuclidin-3-ol with an appropriate anhydride. Quaternization of esters with substituted *meta* and *para*- bromo and methylbenzyl bromides followed. The purity and structure of all compounds were determined by IR, MS, one- and two-dimensional ^1^H- and ^13^C-NMR. Quaternary racemic esters **1**–**12** were then hydrolyzed using horse serum BChE as a catalyst. The hydrolysis was stopped when 50% of the ester was hydrolyzed, Table 1. Acetates, benzoates, isonicotinates and phthalates were hydrolyzed with different rates. Changes to the acyl moiety of the substrate affected the activity of the enzyme: The fastest reactions were those of acetates and isonicotinates, while benzoates and phthalates were hydrolyzed much slower. The best substrate for the enzyme was isonicotinate **10**, while benzoate **8** was the worst substrate. All reactions proceeded slower than the reactions of the appropriate tested non-substituted *N*-benzyl derivatives indicated that *meta* and *para* substituent at the *N*-benzyl protecting group generally lowered the affinity of the enzyme toward the substrates.

After the kinetic resolution, the obtained mixtures of quaternary *N*-benzyl esters and 3-hydroxyquinuclidinium derivatives were subjected to the catalytic transfer hydrogenation, since it was not possible either to monitor optical purity of the reaction directly (e.g., chiral HPLC) or successfully separate quaternary ammonium compounds. Accordingly, chiral (*S*)-quinuclidine esters and (*R*)-quinuclidin-3-ol were isolated after the column chromatography. Specific optical rotation values were determined with an Optical Activity LTD automatic polarimeter and are presented in Table 1. Enantiopurity of the obtained esters or quinuclidin-3-ol for compounds **2**, **6** and **8**–**10** were higher than 95% while the optical purity for other compounds varied from 62–89%. This data imply that BChE is adequate biocatalyst for the kinetic resolution of *N*-benzyl esters: by varying the reaction time, both enantiomers of quinuclidin-3-ol can be obtained in very high optical purity. The enantiopurest (*S*)-esters were benzoates **6** and **8**, compounds with the *para*-bromo and *para*-methyl *N*-benzyl protecting groups. At the same time, the reaction stereoselectivity of derivatives **5** and **7** with *meta* substituents at the benzyl moiety were much lower.

**Table 1:** Hydrolysis of compound **1**–**12** catalyzed with butyrylcholinesterase: optical purities of products were determined after the catalytic transfer hydrogenation.

Compound	Time (BChE hydrolysis) /min	[α]_D_^26^ ester	[α]_D_^26^ quinuclidin-3-ol	Optical purity (*S*)-ester /%	Optical purity (*R*)- quinuclidin-3-ol /%
**1**	80	−140	−80	88	89
**2**	60	−140	−90	88	100
**3**	60	−140	−80	88	89
**4**	60	−140	−80	88	89
**5**	185	−82.3	−60	82	67
**6**	258	−100	−60	100	67
**7**	360	−62	−70	62	78
**8**	1320	−100	−70	100	78
**9**	60	−200	−70	95	78
**10**	45	−200	−70	95	78
**11**	170	110	−70	85	78
**12**	75	100	−80	77	89

**Figure 2:**
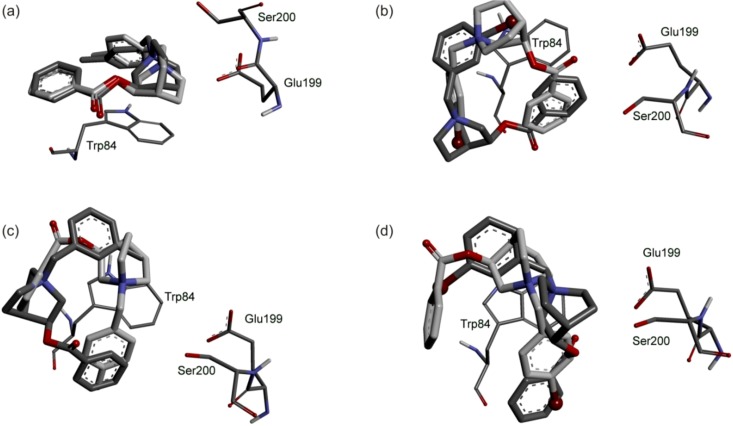
Substrate-BChE complexes derived from docking studies (**a**) **5-*R*** (carbon atoms dark grey) and **6-*R*** (carbon atoms light grey); (**b**) **7-*R*** (carbon atoms dark grey) and **8-*R*** (carbon atoms light grey); (**c**) **5-*S*** (carbon atoms dark grey) and **6-*S*** (carbon atoms light grey); (**d**) **7-*S*** (carbon atoms dark grey) and **8-*S*** (carbon atoms light grey). BChE active site is represented by three structurally important amino acids: Ser200 (part of the catalytic triad), Glu 199 and Trp84 (part of the choline binding site). Hydrogen atoms are omitted for clarity, with the exception of the polar ones.

To identify the binding interactions of the enzyme with the benzoate esters, a molecular docking study was performed using Autodock 4.0.2. The flexible ligands **5**–**8** were docked to the active site of BChE by using the default settings of Lamarckian genetic algorithm. A general trend in binding of all benzoates was observed: Due to the bulkiness of the molecules, productive binding of (*R*)- and (*S*)-esters are not the one with the lowest energies: The carbonyl group of the compounds is pointed far away from Ser200 oxygen atom and the catalytic process cannot occur (Figure 2).

That can explain why the rate of hydrolysis is slower compared to *N*-methyl and non-substituted *N*-benzyl derivatives [11]. Thus, both enantiomers of compounds have to rotate inside the active site for hydrolyses to occur. There is a difference in accommodation of bromo and methyl groups as a result of their different size and electron features. Comparison of *meta-* and *para-*derivatives reveals that position of the substituent determines the modes of binding of substrates: only in the case of **5-*R*** and **6-*R*** (Figure 2a) the position of the methyl substituent does not influence significantly the geometry of the substrate in the most stable complex, while in all other complexes there is a radical change in the position of carbonyl group as well as two aromatic moieties.

## 3. Experimental

### 3.1. General

All reagents and solvents were analytical grade or purified by standard procedures as described in the literature. All melting points were determined on a Melting Point B-540 apparatus (Büchi, Germany) and are uncorrected. IR spectra were recorded with a Perkin-Elmer FTIR 1725 X spectrometer. ^1^H- and ^13^C- 1D and 2D NMR spectra were recorded with a Varian XL-GEM 600 spectrometer at room temperature. Chemical shifts are given in ppm downfield from TMS as internal standard. The reactions with enzyme were carried out in Heidolph UNIMAX 1100 Shaker. BChE (EC 3.1.1.8), type IV-S lyophilized powder from horse serum (Sigma Chemical Co.) was used without further purification. Optical rotations were measured on an Optical Activity AA-10 automatic polarimeter at ambient temperature. Acetate, benzoate, phthalate and isonicotinate esters were synthesized as described previously [12]. Chiral (*R*)- and (*S*)-quinuclidin-3-ols were obtained by the resolution of racemic quinuclidin-3-yl acetates with D-and L-tartaric acid [13]. Standards of chiral alcohol and esters were prepared: (*R*)-quinuclidin-3-ol: [α]_D_^26^ −90° (c = 0.02, 1 M HCl); (*S*)-Quinuclidin-3-yl acetate: [α]_D_^26^ −160° (c = 0.3, CHCl_3_); (*S*)-Quinuclidin-3-yl benzoate: [α]_D_^26^ −100° (c = 0.34, EtOH); (*S*)-Quinuclidin-3-yl isonicotinate: [α]_D_^26^ −211° (c = 0.3, CHCl_3_); (*S*)-Quinuclidin-3-yl phthalate: [α]_D_^26^ 130° (c = 1.0, EtOH).

### 3.2. General Procedure for Quaternization of Esters

The appropriate ester (1.5 mmol) was dissolved in dry acetone. Substituted *N*-benzyl bromide (1.7 mmol) was slowly added to the solution. The mixture was left at room temperature and after 24 h the crystals were formed. They were washed several times with dry ether and dried under reduced pressure to give the title compounds as white crystals (unless stated otherwise).

*3-Acetoyloxy-1-(3-methylbenzyl)quinuclidinium bromide* (**1**). Yield: 41%; m.p. 196.3–197.8 °C; IR (cm^−1^): 2969 (C-H), 2890 (C-H), 1275 (C=O), 1254 (C-O), 1042 (C-N); ^1^H-NMR δ: 1.89–2.05 (m, 2H, H_5_), 2.07 (s, 3H, CH_3_C=O), 2.15–2.23 (m, 2H, H_8_), 2.32–2.35 (s, 3H, CH_3_Bz), 2.38–2.40 (m, 1H, H_4_),3.62–3.78 (m, 1H, H_7_), 3.82–3.89 (m, 2H, H_6_), 3.93–4.04 (m, 1H, H_2b_), 4.24–4.32 (dd, 1H, H_2a_), 5.00–5.10 (m, 3H, H_3_ and CH_2_Bz), 7.23–7.44 (m, 4H, H_2_H_4_H_5_H_6_Bz); ^13^C-NMR δ: 18.39 (C_5_), 21.28 (C_4_), 21.29 (CH_3_Bz), 21,37 (C_8_), 24.69 (CH_3_C=O), 52.87 (C_6_), 53.69 (C_7_), 59.90 (C_2_), 66.70 (CH_2_Bz), 67.16 (C_3_), 126.63 (C_1_Bz), 129.06 (C_5_Bz), 130.45 (C_6_Bz), 131.33 (C_2_Bz), 133.75 (C_4_Bz), 139.13 (C_3_Bz), 170.02 (C=O). ESMS: *m*/*z* (calcd for C_17_H_24_NO_2_^+^ 274.18) found 274.2.

*3-Acetoyloxy-1-(4-methylbenzyl)quinuclidinium bromide* (**2**). Yield: 63%; m.p 174.9–175.6 °C; IR (cm^−1^): 2945 (C-H), 1720 (C=O), 1241 (C-O), 1025 (C-N); ^1^H-NMR δ: 1.89–2.00 (m, 2H, H_5_), 2.08 (s, 3H, CH_3_C=O), 2.14–2.19 (m, 2H, H_8_), 2.33 (s, 3H, CH_3_Bz), 2.35–2.39 (m, 1H, H_4_), 3.62–3.67 (m, 2H, H_7_), 3.83–3.86 (m, 2H, H_6_), 3.93–3.95 (m, 1H, H_2b_), 4.22–4.29 (m, 1H, H_2a_), 4.97–5.07 (m, 3H, H_3_ and CH_2_Bz), 7.15–7.18 (m, 2H, H_3_H_5_Bz), 7.37–7.44 (m, 2H, H_2_H_6_Bz); ^13^C-NMR δ: 17.78 (C_5_), 20.47 (C_4_), 20.76 (CH_3_Bz), 21.37 (C_8_), 24.23 (CH_3_C=O), 52.50 (C_6_), 53.14 (C_7_), 59.36 (C_2_), 65.95 (CH_2_Bz), 66.69 (C_3_), 123.25 (C_1_Bz), 129.25 (C_3_C_5_Bz), 132.71 (C_2_C_6_Bz), 140.13 (C_4_Bz), 169.76 (C=O). ESMS: *m*/*z* (calcd for C_17_H_24_NO_2_^+^ 274.18) found 274.2.

*3-Acetoyloxy-1-(3-bromobenzyl)quinuclidinium bromide* (**3**). Yield: 63%; m.p. 194–196 °C; IR (cm^−1^): 3050, 2955, 2870, 1720, 1430, 1371, 1244, 1026; ^1^H-NMR δ: 1.92–2.03 (m, 2H, H_5_), 2.04–2.09 (s, 3H, CH_3_C=O), 2.18–2.20 (m, 2H, H_8_), 2.39–2.40 (m, 1H, H_4_), 3.70–3.93 (m, 5H, H_6_, H_7_ and H_2b_), 4.33–4.34 (dd, 1H, H_2a_), 5.08–5.21 (m, 3H, H_3_ and CH_2_Bz), 7.36–7.41 (m, 4H, H_2_H_4_ H_5_H_6_Bz); ^13^C-NMR δ: 17.89 (C_5_), 20.51 (C_4_), 20.90 (C_8_), 24.15 (CH_3_C=O), 52.89 (C_6_), 53.34 (C_7_), 59.56 (C_2_), 64.75 (CH_2_Bz), 66.57 (C_3_), 122.58 (C_1_Bz), 128.70 (C_3_Bz), 130.30 (C_6_Bz), 131.80 (C_5_Bz), 133.25 (C_4_Bz), 135.25 (C_2_Bz), 169.67 (C=O). ESMS: *m*/*z* (calcd for C_16_H_21_BrNO_2_^+^ 338.08) found 338.2.

*3-Acetoyloxy-1-(4-bromobenzyl)quinuclidinium bromide* (**4**). Yield: 60%; m.p. 239–241 °C; IR (cm^−1^): 3050, 2956, 2870 (v_s_ C-H); 1735, 1488,1422, 1363, 1239, 1029; ^1^H-NMR δ: 1.89–2.02 (m, 2H, H_5_), 2.04–2.10 (s, 3H, CH_3_C=O), 2.17–2.21 (m, 2H, H_8_), 2.38–2.39 (m, 1H, H_4_), 3.64–3.72 (m, 2H, H_7_), 3.77–3.95 (m, 3H, H_6_ and H_2b_), 4.28–4.29 (dd, 1H, H_2a_), 5.06–5.07 (m, 3H, H_3_), 5.14–5.21 (m, 2H, CH_2_Bz), 7.50–7.52 (m, 2H, H_2_H_6_Bz), 7.56–7.58 (m, 2H, H_3_H_5_Bz); ^13^C-NMR δ: 17.87 (C_5_), 20.47 (C_4_), 20.88 (C_8_); 24.15 (CH_3_C=O), 52.80 (C_6_), 53.21 (C_7_), 59.51 (C_2_), 64.79 (CH_2_Bz), 66.57 (C_3_); 124.91(C_1_Bz), 125.36 (C_4_Bz), 131.96 (C_3_C_5_Bz), 134.47 (C_2_C_6_Bz), 169.65 (C=O). ESMS: *m*/*z* (calcd for C_16_H_21_BrNO_2_^+^ 338.08) found 338.2.

*3-Benzoyloxy-1-(3-methylbenzyl)quinuclidinium bromide* (**5**). Yield: 89%, m.p. 145.8–146.9 °C; IR (cm^−1^): 2969 (C-H), 1710 (C=O), 1453 (C=C), 1278 (C-O), 1024 (C-N); ^1^H-NMR δ: 1.95–2.29 (m, 4H, H_5_ and H_8_), 2.32 (s, 3H, CH_3_Bz), 2.54–2.57 (m, 1H, H_4_), 3.76–4.13 (m, 5H, H_6_, H_7_ and H_2b_), 4.37–4.47 (m, 1H, H_2a_), 5.10 (s, 2H, CH_2_Bz), 5.33–5.36 (m, 1H, H_3_), 7.19–7.29 (m, 2H, H_2_H_6_Bn), 7.39–7.46 (m, 2H, H_2_H_6_Bz), 7.53–7.59 (m, 3H, H_3_H_4_H_5_Bn), 8.01–8.04 (m, 2H, H_4_H_5_Bz); ^13^C-NMR δ: 18. 54 (C_5_), 21.28 (CH_3_Bz), 21.37 (C_8_), 24.94 (C_4_), 53.31 (C_6_), 53.98 (C_7_), 59.97 (C_2_), 66.81 (CH_2_Bz), 67.79 (C_3_), 126.69 (C_1_Bz), 128.81 (C_3_Bz), 129.08 (C_3_C_5_Bn), 128.38 (C_10_), 129.86 (C_2_C_6_Bz), 130.43 (C_2_C_6_Bn), 130.43 (C_5_Bz), 131.29 (C_4_Bz), 133.82 (C_4_Bn), 139.31 (C_3_Bz), 165.40 (C=O). ESMS: *m*/*z* (calcd for C_22_H_26_NO_2_^+^ 336.20) found 336.2.

*3-Benzoyloxy-1-(4-methylbenzyl)quinuclidinium bromide* (**6**). Yield: 81%, m.p. 209.6–210.4 °C; IR (cm^−1^): 2968 (C-H), 2879 (C-H), 1721 (C=O), 1449 (C=C), 1271 (C-O), 1022 (C-N); ^1^H-NMR δ: 1.95–2.28 (m, 4H, H_5_ and H_8_), 2.56 (s, 3H, CH_3_Ph), 2.52–2.61 (m, 1H, H_4_), 3.68–4.16 (m, 5H, H_6_, H_7_ and H_2_), 4.32–4.41 (m, 1H, H_2_), 5.11 (s, 2H, H_16_), 5.32–5.35 (m, 1H, H_3_), 7.18–7.23 (m, 2H, H_11_ and H_15_), 7.43–7.48 (m, 2H, H_18_ and H_22_), 7.51–7.62 (m, 3H, H_12_, H_13_ and H_14_), 8.01–8.04 (m, 2H, H_19_ and H_21_); ^13^C-NMR δ: 18.85 (C_5_), 21.30 (CH_3_Bz), 21.38 (C_8_), 24.98 (C_4_), 53.35 (C_6_), 53.93 (C_7_), 59.99 (C_2_), 66.68 (CH_2_Bz), 67.77 (C_3_), 123.64 (C_4_Bz), 128.61 (C_3_C_5_Bn), 128.76 (C_1_Bz), 129.87 (C_2_C_6_Bz), 129.94 (C_2_C_6_Bn), 133.22 (C_3_C_5_Bz), 133.73 (C_4_Bn), 140.92 (C_1_Bn), 165.51 (C=O). ESMS: *m*/*z* (calcd for C_22_H_26_NO_2_^+^ 336.20) found 336.2.

*3-Benzoyloxy-1-(3-bromobenzyl)quinuclidinium bromide* (**7**). Yield: 78%; m.p. 140–142 °C; IR (cm^−^^1^): 3050, 2966, 2885, 1716, 1450,1409, 1214, 1012; ^1^H-NMR δ: 1.94–2.27 (m, 4H, H_5_ and H_8_), 2.53–2.55 (m, 1H, H_4_), 3.76–4.09 (m, 5H, H_6_, H_7_ and H_2b_), 4.37–4.45 (dd, 1H, H_2a_), 5.10 (s, 2H, CH_2_Bz), 5.33–5.36 (q, 1H, H_3_), 7.24–7.29 (m, 1H, H_4_Bn), 7.38–7.78 (m, 6H, H_2_H_3_H_5_H_6_Bn and H_5_H_6_Bz), 7.99–8.01 (d, 2H, H_2_H_4_Bz); ^13^C-NMR δ: 19.95 (C_5_), 20.10 (C_8_), 24.90 (C_4_), 53.07 (C_6_), 53.58 (C_7_), 55.21 (C_2_), 65.68 (CH_2_Bzl), 67.68 (C_3_), 123.12 (C_1_Bz), 128.60 (C_1_Bn), 129.07 (C_3_C_5_Bn), 129.09 (C_3_Bz), 129.89 (C_2_C_6_Bn), 130.82 (C_4_Bn), 132.30 (C_6_Bz), 133.70 (C_5_Bz), 133.81 (C_4_Bz), 135.72 (C_2_Bz), 165.56 (C=O). ESMS: *m*/*z* (calcd for C_21_H_23_BrNO_2_^+^ 400.09) found 400.2.

*3-Benzoyloxy-1-(4-bromobenzyl)quinuclidinium bromide* (**8**). Yield: 67%; m.p. 84–86 °C; IR (cm^−1^): 3050, 2963, 2885, 1716, 1409, 1214, 1012; ^1^H-NMR δ: 2.03–2.27 (m, 4H, H_5_ and H_8_), 2.55–2.56 (m, 1H, H_4_), 3.72–4.05 (m, 5H, H_6_, H_7_ and H_2b_), 4.35–4.45 (dd, 1H, H_2a_), 5.18 (s, 2H, CH_2_Bz), 5.33–5.36 (q, 1H, H_3_), 7.27–7.29 (m, 1H, H_4_Bn), 7.41–7.59 (m, 6H, H_2_H_3_H_5_H_6_Bn and H_2_H_6_Bz), 7.99–8.01 (d, 2H, H_3_H_5_Bz); ^13^C-NMR δ: 19.39 (C_5_), 20.10 (C_8_), 24.89 (C_4_), 53.07 (C_6_), 53.74 (C_7_), 57.53 (C_2_), 62.16 (CH_2_Bz), 67.73 (C_3_), 125.43 (C_1_Bz), 125.61 (C_1_Bn), 128.62 (C_3_C_5_Bn), 128.70 (C_4_Bz), 128.73 (C_2_C_6_Bz), 129.87 (C_2_C_6_Bn), 131.51 (C_4_Bn), 132.51 (C_3_C_5_Bz), 165.56 (C=O). ESMS: *m*/*z* (calcd for C_21_H_23_BrNO_2_^+^ 400.09) found 400.2.

*3-Isonicotinoyloxy-1-(3-bromobenzyl)quinuclidinium bromide* (**9**). Yield: 78%; m.p. 251–255 °C; IR (cm^−1^): 3050, 2963, 2870, 1720, 1468,1406, 1217, 1039; ^1^H-NMR δ: 1.94–1.95 (m, 2H, H_5_), 2.10–2.12 (m, 2H, H_8_), 2.27–2.31 (m, 1H, H_4_), 3.48–4.19 (m, 5H, H_7_, H_6_ and H_2b_), 4.28–4.30 (dd, 1H, H_2a_), 4.48–4.51 (m, 2H, CH_2_Bz), 5.29–5.30 (q, 1H, H_3_), 7.33 (t, 1H, H_5_Bz), 7.56–7.70 (m, 2H, H_4_H_6_Bz), 7.77–7.78 (m, 1H, H_2_Bz), 7.89–7.90 (m, 2H, H_2_H_6_Py), 8.76–8.77 (m, 2H, H_3_H_5_Py); ^13^C-NMR δ: 17.99 (C_5_), 20.82 (C_8_), 24.32 (C_4_), 52.51 (C_6_), 53.52(C_7_), 59.40 (C_2_), 64.77 (CH_2_Bz); 68.10 (C_3_), 122.52 (C_2_C_6_Py), 122.73 (C_1_Py), 128.52 (C_1_Bz), 130.35 (C_3_C_5_Py), 130.78 (C_6_Bz), 131.33 (C_5_Bz), 133.21 (C_3_Bz), 133.38 (C_2_Bz), 135.23 (C_4_Bz), 135.54 (C=O). ESMS: *m*/*z* (calcd for C_20_H_22_BrN_2_O_2_^+^ 401.09) found 401.1.

*3-Isonicotinoyloxy-1-(4-bromobenzyl)quinuclidinium bromide* (**10**). Yield: 78%; m.p. 119–121 °C; IR (cm^−1^): 3050, 2953, 2870, 1731, 1462,1409, 1214, 1012; ^1^H-NMR δ: 1.92–2.33 (m, 4H, H_5_ and H_8_), 2.57–2.58 (m, 1H, H_4_), 3.50–4.24 (m, 5H, H_6_, H_7_ and H_2b_), 4.32–4.51 (dd, 1H, H_2a_), 5.25 (s, 2H, CH_2_Bz), 5.29–5.41 (q, 1H, H_3_), 7.47–7.66 (m, 4H, H_2_H_3_H_4_H_5_Bz), 7.89–7.91 (m, 2H, H_2_H_6_Py), 8.78–8.80 (m, 2H, H_3_H_5_Py); ^13^C-NMR δ: 18.47 (C_5_), 21.30 (C_8_), 24.05 (C_4_), 53.06 (C_6_), 53.61 (C_7_), 59.78 (C_2_), 65.33 (CH_2_Bz), 68.33 (C_3_), 119.46 (C_2_C_6_Py), 125.69 (C_1_Py), 131.95 (C_2_C_6_Bz), 134.49 (C_3_C_5_Bz), 136.03 (C_1_Bz); 144.13 (C_4_Bz), 151.03 (C_3_C_5_Py), 164.38 (C=O). ESMS: *m*/*z* (calcd for C_20_H_22_BrN_2_O_2_^+^ 401.09) found 401.1.

*3-Phthaloyloxy-1-(3-methylbenzyl)quinuclidinium bromide* (**11**). Yield: 49%, yellow viscous oil; IR (cm^−1^): 3344 (O-H), 2923 (C-H), 1721 (C=O), 1488 (C=C), 1284 (C-O), 1039 (C-N); ^1^H-NMR δ: 0.88–0.99 (m, 2H, H_5_), 1.20–1.48 (m, 2H, H_8_), 1.80–2.15 (m, 1H, H_4_), 2.32–2.40 (CH_3_Bz), 3.38–3.97 (m, 5H, H_6_, H_7_ and H_2b_), 4.65–4.71(m, 1H, H_2a_), 5.17–5.24 (s, 2H, CH_2_Bz), 5.29–5.36 (s, 1H, H_3_), 7.08–7.31 (m, 4H, H_3_H_4_H_5_H_6_Pht), 7.56–8.25 (m, 4H, H_2_H_4_H_5_H_6_Bz); ^13^C-NMR δ: 13.36 (CH_3_Bz), 18.05 (C_5_), 20.62 (C_8_), 21.23 (C_4_), 26.22 (C_6_), 29.98 (C_7_), 64.05 (C_3_), 67.53 (CH_2_Bz), 67.83 (C_2_), 125.49 (C_2_Bz), 128.41–129.29 (C_2_C_4_C_5_C_6_Bz), 130.55 (C_1_Pht), 130.89–131.97 (C_3_C_4_C_5_C_6_Pht), 132.75 (C_1_Bz), 135.17 (C_2_Pht), 138.35 (C_3_Bz), 168.09 (COOH), 171.03 (C=O). ESMS: *m*/*z* (calcd for C_23_H_26_NO_4_^+^ 380.19) found 380.3.

*3-Phthaloyloxy-1-(4-methylbenzyl)quinuclidinium bromide* (**12**). Yield: 81%, yellow viscous oil; IR (cm^−1^): 3419 (O-H), 2957 (C-H), 1722 (C=O), 1458 (C=C), 1285 (C-O), 1039 (C-N); ^1^H-NMR δ: 1.77–1.87 (m, 4H, H_5_ and H_8_), 2.27–2.35 (m, 1H, H_4_), 2.28 (s, 3H, CH_3_Bz), 3.45–3.52 (m, 5H, H_6_, H_7 _and H_2b_), 3.94–3.90 (m, 1H, H_2a_), 4.84 (s, 2H, CH_2_Bz), 4.51 (q, 1H, H_3_); 7.11–7.16 (m, 4H, H_2_H_3_H_5_H_6_Bz), 7.27–7.36 (m, 2H, H_4_H_5_Pht), 7.45–7.52 (m, 2H, H_3_H_6_Pht), 8.19–8.24 (m, 1H, COOH); ^13^C-NMR δ: 17.97 (C_5_), 21.23 (C_4_), 21.57 (C_8_), 26.92 (CH_3_Bz), 53.27 (C_7_), 54.36 (C_6_), 63.77 (C_2_), 64.09 (C_3_), 67.63 (CH_2_Bz), 123.44 (C_1_Bz), 129.93 (C_3_C_6_Pht), 131.04 (C_2_C_3_C_5_C_6_Bz), 132.43 (C_4_Pht), 132.88 (C_5_Pht), 134.11 (C_1_C_2_Pht), 140.89 (C_4_Bz), 170.95 (C=O, COOH). ESMS: *m*/*z* (calcd for C_23_H_26_NO_4_^+^ 380.19) found 380.3.

### 3.3. Kinetic Resolution with BChE

The appropriate quaternary ester (40 mg) was dissolved in a minimal volume of 0.1 M phosphate buffer. The solution was placed in shaker for 10 min and BChE (100 μL, 10 mg/mL) was added to a reaction mixture. Reaction was stopped when 50% of the ester was hydrolyzed by adding ethanol (10 mL). The reaction mixture was dried under the reduced pressure and extracted with chloroform. The extract was dried over Na_2_SO_4_ and evaporated under reduced pressure.

### 3.4. Catalytic Transfer Hydrogenation

Dry methanol (3 mL) was added to the residue. This methanol solution was placed in a 2-bottom flask and 10% Pd-C (40 mg) was added. Ammonium formate (30 mg) was added in a single portion. The reaction mixture was stirred at the reflux temperature. After the completion of the reaction, it was filtered through a Celite pad, and washed with dry methanol (5 mL). The filtrate was dried under the reduced pressure, made alkaline with saturated aqueous solution of K_2_CO_3_ and extracted with chloroform. The extract was dried and evaporated under reduced pressure. Products were separated by column chromatography (aluminum oxide 90 active neutral (70–230 mesh ASTM), (Merck, Darmstadt, Germany) with chloroform-methanol solution (9:1) as eluent.

## 4. Conclusions

A series of novel *meta-* and *para-*substituted *N*-benzyl protected quinuclidinium esters were prepared and stereoselectivity of hydrolysis catalyzed with horse serum BChE was investigated. The kinetic resolutions were performed at 37 °C in 0.1 M phosphate buffer, pH 7.4. The hydrolyses of (*R*)-esters proceeded much faster than those of (*S*)-enantiomers. Introduction of *N*-benzyl *para-* and *meta-* bromine atoms or methyl groups resulted in a significant improvement of the stereoselectivity compared to non-substituted *N*-benzyl protected groups. Thus, optically pure quinuclidin-3-ol derivatives were prepared in high yields and enantiopurity by the esterification, quaternization with derivatives of benzyl bromide, BChE-catalysed hydrolysis and finally catalytic transfer hydrogenation.
